# Classification of a Massive Number of Viral Genomes and Estimation of Time of Most Recent Common Ancestor (tMRCA) of SARS-CoV-2 Using Phylodynamic Analysis

**DOI:** 10.21769/BioProtoc.4955

**Published:** 2024-03-20

**Authors:** Xiaowen Hu, Siqin Guan, Yiliang He, Guohui Yi, Lei Yao, Jiaming Zhang

**Affiliations:** 1 Key Laboratory of Microbiology of Hainan, Institute of Tropical Bioscience and Biotechnology, Chinese Academy of Tropical Agricultural Sciences, Haikou, China; 2 Institute of South Subtropical Crops, Chinese Academy of Tropical Agricultural Sciences, Zhanjiang, China; 3 College of Animal Sciences, Huazhong Agricultural University, Wuhan, China; 4 Public Research Laboratory, Hainan Medical University, Haikou, China; 5 Sichuan Provincial Key Laboratory for Human Disease Gene Study and the Center for Medical Genetics, Department of Laboratory Medicine, Sichuan Academy of Medical Sciences & Sichuan Provincial People's Hospital, University of Electronic Science and Technology, Chengdu, China

**Keywords:** Viral origin, Genome classification, Genetic linkage, Bayesian phylodynamic analysis, SARS-CoV-2, Redundant genome, Diversity

## Abstract

Estimating the time of most recent common ancestor (tMRCA) is important to trace the origin of pathogenic viruses. This analysis is based on the genetic diversity accumulated in a certain time period. There have been thousands of mutant sites occurring in the genomes of SARS-CoV-2 since the COVID-19 pandemic started; six highly linked mutation sites occurred early before the start of the pandemic and can be used to classify the genomes into three main haplotypes. Tracing the origin of those three haplotypes may help to understand the origin of SARS-CoV-2. In this article, we present a complete protocol for the classification of SARS-CoV-2 genomes and calculating tMRCA using Bayesian phylodynamic method. This protocol may also be used in the analysis of other viral genomes.

Key features

• Filtering and alignment of a massive number of viral genomes using custom scripts and ViralMSA.

• Classification of genomes based on highly linked sites using custom scripts.

• Phylodynamic analysis of viral genomes using Bayesian evolutionary analysis sampling trees (BEAST).

• Visualization of posterior distribution of tMRCA using Tracer.v1.7.2.

• Optimized for the SARS-CoV-2.

**Graphical overview**


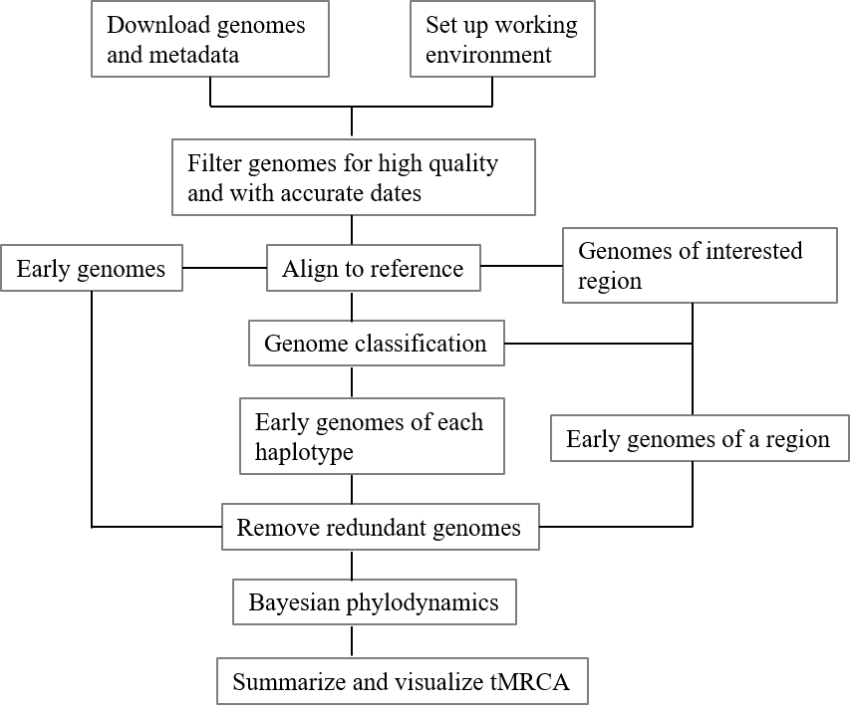


**Graphical workflow of time of most recent common ancestor (tMRCA) estimation process**

## Background

Revealing the origins of pathogenic viruses, crucial for cutting them off from the root and preventing future spillover, requires long-term hard work from scientists all around the world [1]. Although some infectious pathogens can be traced back decades, the debate on their origin continues. For example, AIDS was officially reported on June 5, 1981, by the Centers for Disease Control and Prevention of the USA. Five years later, HIV infection was detected in a human serum sample collected in Léopoldville in early 1959 [2]. Bayesian phylodynamic analyses using recovered viral gene sequences from decades-old paraffin-embedded tissues traced the most recent common ancestor (MRCA) of the M group of HIV back to approximately 1908 (CI 1884–1924), suggesting that HIV has been circulating in the human population for approximately 100 years [3]. MERS-CoV is another example, as it was first reported in a Saudi Arabian man in 2012 [4]. Bats are thought to be the reservoir hosts of MERS-CoV, and dromedary camels are considered to be the major intermediate host [5]; however, the transmission route from animals to humans is not well understood. Researchers tested 189 camel serum samples from 1983 to 1997 and found that 81% had neutralizing antibodies against MERS-CoV, suggesting long-term virus circulation in these animals [6]. Similarly, COVID-19 was first reported on December 27, 2019, in Wuhan, China [7,8], and the Huanan seafood market was suspected to be the place of origin [9]; however, disputes remain. Pekar and colleagues explored the evolutionary dynamics of the first wave of SARS-CoV-2 infections in China using a strict clock Bayesian phylodynamic analysis but failed to capture the index case [10], probably because the redundant sequences were not removed, which usually influences the accuracy of time of MRCA (tMRCA) estimation, as indicated in two recent tMRCA analysis [11,12]. Genome classification plays a critical role in tracing the origin of pathogenic viruses [3,12]. We have previously classified SARS-CoV-2 genomes based on two amino acids, Spike-614 and Orf8-84, and revealed 16 haplotypes. From those, three major haplotypes were found to separately drive the development of the pandemic in China and the world. However, genome classification based on amino acid mutations did not rule out recombination and reverse mutations. In this paper, we provide detailed protocols to filter and classify the massive number of viral genomes according to six highly linked mutations that happened in the early phase of the epidemic by custom scripts and common programs and estimate tMRCA of non-redundant genome subpools.

## Materials and reagents

SARS-CoV-2 genome sequences were obtained from the GISAID database [13] on May 1, 2022. Genomes that were collected from hosts other than humans and/or had a length of less than 2,900 nucleotides and more than 0.05% of unknown nucleotides were filtered out. More than 5 million genomes were retained for further analysis.

## Equipment

1. Bioinformatic platform (CentOS, 7.2.1511)
2. Windows desktop computers (v11, 6/6/2021)

## Software and datasets

1. Perl (v5.34.0, 20/5/2021)
2. Python (v3.9.0, 5/10/2020)
3. Gcc (v8.4.1, 28/9/2020)
4. SeqKit (v2.3.0, 12/8/2022)
5. minimap2 (v2.24, 26/12/2021)
6. ViralMSA (v3, 29/6/2007)
7. cd-hit (v4.8.1, 1/9/2018)
8. ALTER (v1.3.4, 30/10/2016)
9. BEAST (v1.10.4, 9/11/2018)
10. Jdk (v17_windows-x64_bin, 16/6/2021)
11. Jre (8u341-windows-x64, 5/6/2021)
12. Tracer (v1.7.2, 5/1/2018)
13. Global Initiative of Sharing All Influenza Data (GISAID) (https://gisaid.org/) (Access date, 1/5/2022)

**Figure 1.**
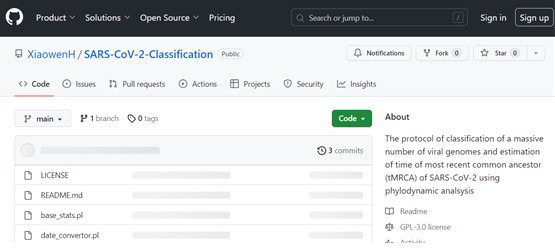
Screenshot of the personalized scripts deposited in GitHub

All personalized scripts have been deposited in GitHub: https://github.com/XiaowenH/SARS-CoV-2-Classification (Access date, 5/9/2023). Figure 1 is a screenshot of the files in GitHub.

## Procedure

- **Set up the working environment**
  1. Create a Python environment of conda.
$ wget https://repo.anaconda.com/miniconda/Miniconda3-latest-Linux-x86_64.sh
    $ bash Miniconda3-latest-Linux-x86_64.sh -b -p ~/miniconda
    $ export PATH="/$HOME/miniconda/bin:$PATH"
    $ conda create -n myenv python=3.9
    $ conda activate myenv
    #Please note that "$HOME" corresponds to the PATH you want to install in the conda environment (the same hereafter).
  2. Install the following software:
    - Install Seqkit in a Linux platform.
      $ conda install -c bioconda seqkit
    - Install minimap2 in a Linux platform.
      $ git clone https://github.com/lh3/minimap2
      $ cd minimap2 && make
    - Install ViralMSA [14] in a Linux platform.
$ wget "https://raw.githubusercontent.com/niemasd/ViralMSA/master/ViralMSA.py"
      $ chmod a+x ViralMSA.py
      $ sudo mv ViralMSA.py /usr/local/bin/ViralMSA.py
    - Install CD-HIT in a Linux platform.
      $ git clone https://github.com/weizhongli/cdhit.git
      $ cd cd-hit
      $ make
      $ cd cd-hit-auxtools
      $ make
    - Install Alter in a Linux platform.
      $ git clone https://github.com/sing-group/ALTER.git
      $ cd ALTER
      $ /$HOME/apache-maven-3.8.4/bin/mvn
    - Install BEAST [15] in a Windows platform.
      # Download BEAST.v1.10.4 at
      http://beast.community/programs
    - Install BEAGLE in a Windows platform.
      # Download BEAGLE at
      https://github.com/beagle-dev/beagle-lib
    - Install Tracer v1.7.2 in a Windows platform.
      # Download BEAGLE at
      http://beast.community/tracer
- **Download the viral genomes and metadata**
  1. Download SARS-CoV-2 genomes and meta.tsv files from the GISAID database [16] after login (https://gisaid.org/). Note that the content in each column in the meta.tsv may change (e.g., the accession numbers were put in column 3 in the metadata downloaded on May 1, 2022, but in column 5 in the metadata downloaded on May 1, 2023).
  2. Unpack the files.
    $ xz -d sequences_fasta_2022_04_29.tar.xz
    $ tar -xvf sequences_fasta_2022_04_29.tar
    $ xz -d metadata_tsv_2022_04_29.tar.xz
    $ tar -xvf metadata_tsv_2022_04_29.tar
- **Rename the taxa in the Fasta file of the genomes by accession numbers (e.g., EPI_ISL_4405694) using a custom script**
  $ perl rename_fasta_taxa_to_tsv_acc_column3.pl -t meta.tsv -f sequence.fasta
- **Fetch GISAID reference sequence**
  1. Create a file named “accession.txt,” paste in EPI_ISL_402124, and save.
    $ echo ‘EPI_ISL_402124’ >accession.txt
    # EPI_ISL_402124 is the reference sequence used by the GISAID database and in many researches.
  2. Fetch the reference sequence from total sequence file.
    $ cat sequence.fasta | /$HOME/seqkit grep -f accession.txt -t dna -j 10 -o reference_wiv04.fasta
- **Retrieve genomes with complete, high coverage sequences and accurate dates and sampled from human hosts**
  The accession number, host, completeness, and coverage of the genomes are located in columns 3, 8, 18, and 19, respectively, in the metadata of April 29, 2022. The sample collection date is located in column 4. The column number may be different in the metadata downloaded at a different day.
  1. Filter metadata.tsv for accessions with complete genomes, with high coverage, and from human hosts.
    $ awk -F '\t' '{if($18 == "True" && $19 == "True" && $8 == "Human") print}' metadata.tsv >global_2022_04_29_human_complete_metadata.tsv
  2. Filter metadata.tsv for accessions with accurate sample collection date using a custom script: dates_filter.pl.
    $ perl dates_filter.pl -t global_2022_04_29_human_complete_metadata.tsv -o global_2022_04_29_human_complete_dates_metadata.tsv
  3. Print selected accession numbers to a file from metadata.
    $ awk -F '\t' '{print $3}' global_2022_04_29_human_complete_dates_metadata.tsv > global_2022_04_29_human_complete_dates_accessions.txt
  4. Retrieve genome sequences with complete and high coverage and accurate dates.
    $ cat sequence.fasta | /$HOME/seqkit grep -f global_2022_04_29_human_complete_dates_accessions.txt -t dna -j 10 -o global_2022_04_29_human_complete_dates_genome.fasta
- **Align the genome sequences**
  $ /$HOME/ViralMSA-master/ViralMSA.py -e [your email] -s global_2022_04_29_human_complete_dates_genome.fasta -o output -r reference_wiv04.fasta --omit_ref
- **All genome classification**
  1. Retrieve the six highly linked sites using the custom script fetch_nucleotides_from_alignments.pl (Figure 2).
    $ perl fetch_nucleotides_from_alignments.pl -f global_2022_04_29_human_complete_dates_genome.fasta.aln
    -r 241-241,3037-3037,8782-8782,14408-14408,23403-23403,28144-28144 -o global_2022_04_29_human_complete_dates_genome.fasta_six_sites.txt
    **Figure 2.**
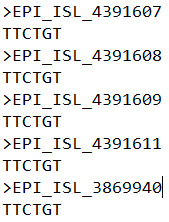
Output format of the six linked sites in the genomes. The six nucleotides of sites 241, 3037, 8782, 14408, 23403, and 28144 were retrieved from SARS-CoV-2 genomes aligned to reference genome wiv04 EPI_ISL_402124.
  2. Classify the genomes into haplotypes by the six linked mutation sites (Figure 3 ).
    $ cat global_2022_04_29_human_complete_dates_genome.fasta_six_sites.txt | /$HOME/seqkit grep -s -i –p CCTCAC > global_2022_04_29_human_complete_dates_DS.txt
    $ cat global_2022_04_29_human_complete_dates_genome.fasta_six_sites.txt | /$HOME/seqkit grep -s -i -p CCCCAT > global_2022_04_29_human_complete_dates_DL.txt
    $ cat global_2022_04_29_human_complete_dates_genome.fasta_six_sites.txt | /$HOME/seqkit grep -s -i -p TTCTGT > global_2022_04_29_human_complete_dates_GL.txt
    $ /$HOME/seqkit stats global_2022_04_29_human_complete_dates_DS.txt >haplotype.statistics.txt
    $ /$HOME/seqkit stats global_2022_04_29_human_complete_dates_DL.txt >>haplotype.statistics.txt
    $ /$HOME/seqkit stats global_2022_04_29_human_complete_dates_GL.txt >>haplotype.statistics.txt
    **Figure 3.**
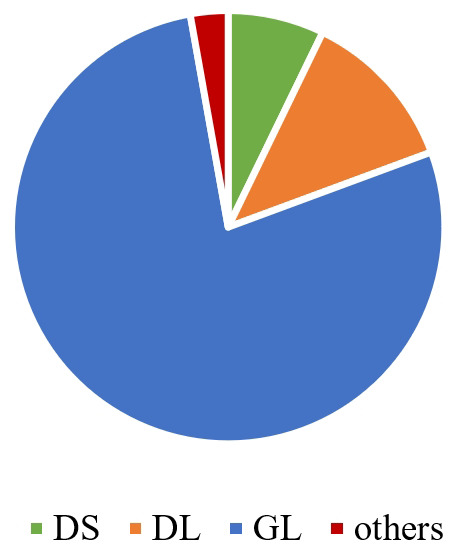
Pie chart of haplotypes in global SARS-CoV-2 genomes. DS (CCTCAC), DL (CCCCAT), and GL (TTCTGT) are the three main haplotypes classified by the six linked sites.
  3. Fetch accession numbers of each haplotype.
    $ /$HOME/seqkit seq global_2022_04_29_human_complete_dates_DS.txt -n > global_DS.accessions.txt
    $ /$HOME/seqkit seq global_2022_04_29_human_complete_dates_DL.txt -n > global_DL.accessions.txt
    $ /$HOME/seqkit seq global_2022_04_29_human_complete_dates_GL.txt -n > global_GL.accessions.txt
  4. Retrieve aligned genome sequences of each haplotype.
    $ cat global_2022_04_29_human_complete_dates_genome.fasta.aln | /$HOME/seqkit grep -f global_DS.accessions.txt -t dna -j 10 -o global_DS.aln
    $ cat global_2022_04_29_human_complete_dates_genome.fasta.aln | /$HOME/seqkit grep -f global_DL.accessions.txt -t dna -j 10 -o global_DL.aln
    $ cat global_2022_04_29_human_complete_dates_genome.fasta.aln | /$HOME/seqkit grep -f global_GL.accessions.txt -t dna -j 10 -o global_GL.aln
- **Classification of early genomes collected in the early phase of the pandemic (from beginning to end of April 2020)**
  The sample collection date is located in column 4 in the metadata of April 29, 2022.
  1. Retrieve accession numbers of early genomes.
    $ awk -F '\t' '{if($4~/2019-12/) print}' global_2022_04_29_human_complete_dates_metadata.tsv > global_early_meta.tsv
    $ awk -F '\t' '{if($4~/2020-01/) print}' global_2022_04_29_human_complete_dates_metadata.tsv >> global_early_meta.tsv
    $ awk -F '\t' '{if($4~/2020-02/) print}' global_2022_04_29_human_complete_dates_metadata.tsv >> global_early_meta.tsv
    $ awk -F '\t' '{if($4~/2020-03/) print}' global_2022_04_29_human_complete_dates_metadata.tsv >> global_early_meta.tsv
    $ awk -F '\t' '{if($4~/2020-04/) print}' global_2022_04_29_human_complete_dates_metadata.tsv >> global_early_meta.tsv
    $ awk -F '\t' '{print $3}' global_early_meta.tsv >global_early_accessions.txt
  2. Retrieve the aligned sequences of the early genomes.
    $ cat global_2022_04_29_human_complete_dates_genome.fasta.aln | /$HOME/seqkit grep -f global_early_accessions.txt -t dna -j 10 -o global_early_all_haplotype.fasta.aln
  3. Retrieve the six highly linked sites using the custom script fetch_nucleotides_from_alignments.pl.
    $ perl fetch_nucleotides_from_alignments.pl -f global_early_all_haplotype.fasta.aln
    -r 241-241,3037-3037,14408-14408,23403-23403,28144-28144 -o global_early.aln_six_sites.txt
  4. Classify the genomes.
    $ cat global_early.aln_six_sites.txt | /$HOME/seqkit grep -s -i -p CCTCAC > global_early_DS.txt
    $ cat global_early.aln_six_sites.txt | /$HOME/seqkit grep -s -i -p CCCCAT > global_early_DL.txt
    $ cat global_early.aln_six_sites.txt | /$HOME/seqkit grep -s -i -p TTCTGT > global_early_GL.txt
  5. Fetch accession numbers of each haplotype.
    $ /$HOME/seqkit seq global_early_DS.txt -n > global_early_DS.accessions.txt
    $ /$HOME/seqkit seq global_early_DL.txt -n > global_early_DL.accessions.txt
    $ /$HOME/seqkit seq global_early_GL.txt -n > global_early_GL.accessions.txt
  6. Retrieve aligned genome sequences of each haplotype.
    $ cat global_early_all_haplotype.fasta.aln | /$HOME/seqkit grep -f global_ early_DS.accessions.txt -t dna -j 10 -o global_ early_DS.aln
    $ cat global_early_all_haplotype.fasta.aln | /$HOME/seqkit grep -f global_ early_DL.accessions.txt -t dna -j 10 -o global_ early_DL.aln
    $ cat global_early_all_haplotype.fasta.aln | /$HOME/seqkit grep -f global_ early_GL.accessions.txt -t dna -j 10 -o global_ early_GL.aln
- **Bayesian phylodynamic analysis using the early genomes of three haplotypes as examples**
  1. Filter out genomes with unknown higher than 0.05%.
    - Filter out genomes of DS (CCTCAC) haplotypes with unknown nucleotides higher than 0.05%.
      # Calculate nucleotide composition of each sequence in each haplotype
      $ perl base_stats.pl global_early_DS.aln
      # Pick up accessions with unknowns < 0.05%
      $ awk -F '\t' '{if($6 < 0.0005) print$1}' global_early_DS.aln.stats > global_early_DS.aln_0.0005N_ID.txt
      # Fetch aligned genome sequences with unknowns <0.05%
      $ cat global_early_DS.aln |/$HOME/seqkit grep -f global_early_DS.aln_0.0005N_ID.txt -t dna -j 10 -o global_early_DS_0.0005N.aln
    - Filter out genomes of DL (CCCCAT) haplotypes with unknown nucleotides higher than 0.05%.
      # Calculate nucleotide composition of each sequence in each haplotype
      $ perl base_stats.pl global_early_DL.aln
      # Pick up accessions with unknowns < 0.05%
      $ awk -F '\t' '{if($6 < 0.0005) print$1}' global_early_DL.aln.stats > global_early_DL.aln_0.0005N_ID.txt
      # Fetch aligned genome sequences with unknowns <0.05%
      $ cat global_early_DL.aln |/$HOME/seqkit grep -f global_early_DL.aln_0.0005N_ID.txt -t dna -j 10 -o global_early_DL_0.0005N.aln
    - Filter out genomes of GL (TTCTGT) haplotypes with unknown nucleotides higher than 0.05%.
      # Calculate nucleotide composition of each sequence in each haplotype.
      $ perl base_stats.pl global_early_GL.aln
      # Pick up accessions with unknowns < 0.05%
      $ awk -F '\t' '{if($6 < 0.0005) print$1}' global_early_GL.aln.stats > global_early_GL.aln_0.0005N_ID.txt
      # Fetch aligned genome sequences with unknowns <0.05%
      $ cat global_early_GL.aln | /$HOME/seqkit grep -f global_early_GL.aln_0.0005N_ID.txt -t dna -j 10 -o global_early_GL_0.0005N.aln
    - Remove redundant genomes with a threshold of 0.9997.
      $ /$HOME/cd-hit-v4.8.1-2019-0228/cd-hit-est -i global_early_DS_0.0005N.aln -o global_early_DS_0.0005N_CDHit0.9997.fas -M 2500 -c 0.9997 -aL 1 -aS 1 -d 0
      $ /$HOME/cd-hit-v4.8.1-2019-0228/cd-hit-est -i global_early_DL_0.0005N.aln -o global_early_DL_0.0005N_CDHit0.9997.fas -M 2500 -c 0.9997 -aL 1 -aS 1 -d 0
      $ /$HOME/cd-hit-v4.8.1-2019-0228/cd-hit-est -i global_early_GL_0.0005N.aln -o global_early_GL_0.0005N_CDHit0.9997.fas -M 2500 -c 0.9997 -aL 1 -aS 1 -d 0
    - Change format to NEX.
      $ java -jar /$HOME/ALTER/alter-lib/target/ALTER-1.3.4-jar-with-dependencies.jar -i global_early_DS_0.0005N_CDHit0.9997.fas -ia -o global_early_DS_0.0005N_CDHit0.9997.fas.nex -of NEXUS -oo Windows -op MrBayes
      $ java -jar /$HOME/ALTER/alter-lib/target/ALTER-1.3.4-jar-with-dependencies.jar -i global_early_DL_0.0005N_CDHit0.9997.fas -ia -o global_early_DL_0.0005N_CDHit0.9997.fas.nex -of NEXUS -oo Windows -op MrBayes
      $ java -jar /$HOME/ALTER/alter-lib/target/ALTER-1.3.4-jar-with-dependencies.jar -i global_early_GL_0.0005N_CDHit0.9997.fas -ia -o global_early_GL_0.0005N_CDHit0.9997.fas.nex -of NEXUS -oo Windows -op MrBayes
    - Fetch dates for accessions from metadata.
      $ awk -F "\t" -v OFS="\t" '{print $3,$4}' global_early_meta.tsv > global_early_dates.txt
    - Convert dates to decimal dates using a custom script date_convertor.pl.
      $ perl date_convertor.pl global_early_dates.txt > global_early_decimal_dates
    - Create BEAST XML file in Windows platform.
      # Open BEAUti-v1.10.4 by double-click its the icon
      # Load genome alignment file in NEX format
      # Import dates (sample collection dates) in the file global_early_decimal_dates
      # Set options for analysis, e.g. set ‘Clocks’ to either strict, or relaxed, set ‘Tree Prior’ to either ‘Bayesian Skyline’ or ‘Bayesian SkyGrid’, set MCMC ‘Length of Chain’ to 1,200 million generations. The run may be stopped when the Explained Sum of Squares (ESS) of all parameters are significant (>200). When the parameters are all set, click ‘Generate BEAST file’.
    - Run BEAST.
      # Open BEAST v1.10.4 by double-click the icon
      # Load BEAST XML file, click ‘Run’. The run may take a few days until all ESSs are significant.
    - Visualize tMRCA by Tracer (Figure 4).
      # Open Tracer v1.7.2 by double click the icon
      # Import the log file created by BEAST. Posterior distribution of tMRCA can be shown by clicking ‘age(root)’ and ‘Marginal Density’. The mean tMRCA and 95% HPD interval are provided in ‘Estimates’.
  **Figure 4.**
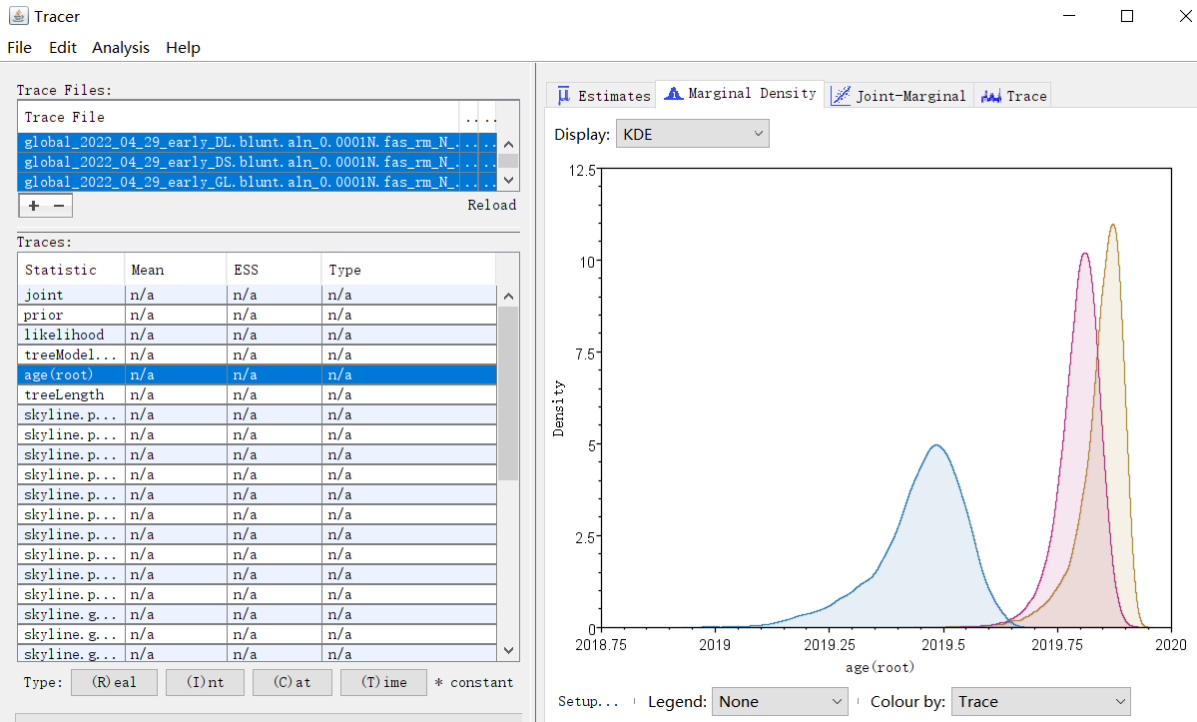
Screenshot of time of most recent common ancestor (tMRCA) estimation of three main haplotypes of the early SARS-CoV-2 genomes as summarized with Tracer. The dates are shown in decimal.

## Validation of protocol

This protocol or parts of it has been used and validated in the following research articles:

- Hu et al. [17]. Genome characterization based on the Spike-614 and NS8-84 loci of SARS-CoV-2 reveals two major possible onsets of the COVID-19 pandemic. *PLoS ONE* 18(6): e0279221. https://doi.org/10.1371/journal.pone.0279221 (Figure 4, panel 1; Figure 6).
- Guan et al. [12]. Genome analysis reveals much earlier separation and parallel evolution of major haplotypes of SARS-CoV-2 than its occurrence in China. *Science in One Health* 2(2023), https://doi.org/10.1016/j.soh.2023.100041 (Figure 1; Figure 3; Figure 4).

## References

[1] Tong Y., Liu W., Liu P., Liu W. J., Wang Q. and Gao G. F. (2021). The origins of viruses: discovery takes time, international resources, and cooperation. Lancet 398(10309): 1401-1402. 34600605 10.1016/S0140-6736(21)02180-2 PMC8483647

[2] Nahmias A., Weiss J., Yao X., Lee F., Kodsi R., Schanfield M., Matthews T., Bolognesi D., Durack D., Motulsky A., .(1986). Evidence for human infection with an HTLV III/LAV-like virus in Central Africa, 1959. Lancet 327(8492): 1279-1280. 10.1016/s0140-6736(86)91422-4 2872424

[3] Worobey M., Gemmel M., Teuwen D. E., Haselkorn T., Kunstman K., Bunce M., Muyembe J. J., Kabongo J. M., Kalengayi R. M., Van Marck E., .(2008). Direct evidence of extensive diversity of HIV-1 in Kinshasa by 1960. Nature 455 (7213): 661-664. 18833279 10.1038/nature07390 PMC3682493

[4] Zaki A. M., van Boheemen S., Bestebroer T. M., Osterhaus A. D. and Fouchier R. A. (2012). Isolation of a Novel Coronavirus from a Man with Pneumonia in Saudi Arabia. N. Engl. J. Med . 367(19): 1814-1820 . 23075143 10.1056/NEJMoa1211721

[5] Azhar E. I., El-Kafrawy S. A., Farraj S. A., Hassan A. M., Al-Saeed M. S., Hashem A. M. and Madani T. A. (2014). Evidence for Camel-to-Human Transmission of MERS Coronavirus. N. Engl. J. Med. 370 (26): 2499-2505. 24896817 10.1056/NEJMoa1401505

[6] Müller M. A., Corman V. M., Jores J., Meyer B., Younan M., Liljander A., Bosch B. J., Lattwein E., Hilali M., Musa B. E., .(2014). MERS Coronavirus Neutralizing Antibodies in Camels, Eastern Africa, 1983–1997. Emerging Infectious Diseases 20(12): 2093 -2095. 25425139 10.3201/eid2012.141026 PMC4257824

[7] Zhu N., Zhang D., Wang W., Li X., Yang B., Song J., Zhao X., Huang B., Shi W., Lu R., .(2020). A Novel Coronavirus from Patients with Pneumonia in China, 2019. N. Engl. J. Med. 382(8): 727-733. 31978945 10.1056/NEJMoa2001017 PMC7092803

[8] Wu F., Zhao S., Yu B., Chen Y. M., Wang W., Song Z. G., Hu Y., Tao Z. W., Tian J. H., Pei Y. Y., .(2020). A new coronavirus associated with human respiratory disease in China. Nature 579 (7798): 265-269. 32015508 10.1038/s41586-020-2008-3 PMC7094943

[9] Li Q., Guan X., Wu P., Wang X., Zhou L., Tong Y., Ren R., Leung K. S., Lau E. H., Wong J. Y., .(2020). Early Transmission Dynamics in Wuhan, China, of Novel Coronavirus–Infected Pneumonia. N. Engl. J. Med. 382(13): 1199 -1207. 31995857 10.1056/NEJMoa2001316 PMC7121484

[10] Pekar J., Worobey M., Moshiri N., Scheffler K. and Wertheim J. O. (2021). Timing the SARS-CoV-2 index case in Hubei province. Science 372( 6540): 412-417. 33737402 10.1126/science.abf8003 PMC8139421

[11] Cheng C. and Zhang Z. (2023). SARS-CoV-2 shows a much earlier divergence in the world than in the Chinese mainland. Sci. China Life Sci. 66(6): 1440- 1443. 36897494 10.1007/s11427-023-2294-5 PMC9999321

[12] Guan S., Hu X., Yi G., Yao L. and Zhang J. (2023). Genome analysis of SARS-CoV-2 haplotypes: separation and parallel evolution of the major haplotypes occurred considerably earlier than their emergence in China. Science in One Health 2: 100041. 10.1016/j.soh.2023.100041 PMC11262268 39077033

[13] Khare S., Gurry C., Freitas L., Schultz M. B., Bach G., Diallo A., Akite N., Ho J., Lee R. T., Yeo W., .(2021). GISAID's role in pandemic response . China CDC Wkly 3(49): 1049-1051. 34934514 10.46234/ccdcw2021.255 PMC8668406

[14] Moshiri N. (2020). ViralMSA: massively scalable reference-guided multiple sequence alignment of viral genomes. Bioinformatics(Oxford, England) 37(5): 714- 716. 10.1093/bioinformatics/btaa743 32814953

[15] Suchard M. A., Lemey P., Baele G., Ayres D. L., Drummond A. J. and Rambaut A. (2018). Bayesian phylogenetic and phylodynamic data integration using BEAST 1.10. Virus Evol. 4(1): vey016. 29942656 10.1093/ve/vey016 PMC6007674

[16] Shu Y. and McCauley J. (2017). GISAID: Global initiative on sharing all influenza data– from vision to reality. Eurosurveillance 22(13): e30494. 10.2807/1560-7917.ES.2017.22.13.30494 PMC5388101 28382917

[17] Hu X., Mu Y., Deng R., Yi G., Yao L. and Zhang J. (2023). Genome characterization based on the Spike-614 and NS8-84 loci of SARS-CoV-2 reveals two major possible onsets of the COVID-19 pandemic. PLoS One 18( 6): e0279221. 37319292 10.1371/journal.pone.0279221 PMC10270620

